# Parts list for a microtubule depolymerising kinesin

**DOI:** 10.1042/BST20180350

**Published:** 2018-11-22

**Authors:** Claire T. Friel, Julie P. Welburn

**Affiliations:** 1 School of Life Sciences, University of Nottingham, Medical School, QMC, Nottingham NG7 2UH, U.K.; 2 Wellcome Trust Centre for Cell Biology, University of Edinburgh, Michael Swann Building, The King's Buildings, Mayfield Road, Edinburgh EH9 3BF, U.K.

**Keywords:** cytoskeleton, depolymerising, kinesins, kinesin-13, microtubule, molecular motors

## Abstract

The Kinesin superfamily is a large group of molecular motors that use the turnover of ATP to regulate their interaction with the microtubule cytoskeleton. The coupled relationship between nucleotide turnover and microtubule binding is harnessed in various ways by these motors allowing them to carry out a variety of cellular functions. The Kinesin-13 family is a group of specialist microtubule depolymerising motors. Members of this family use their microtubule destabilising activity to regulate processes such as chromosome segregation, maintenance of cilia and neuronal development. Here, we describe the current understanding of the structure of this family of kinesins and the role different parts of these proteins play in their microtubule depolymerisation activity and in the wider function of this family of kinesins.

## Kinesin-13s are a family of specialist microtubule depolymerisers

The Kinesin superfamily of molecular motors uses the turnover of ATP to regulate their interaction with the microtubule cytoskeleton. The superfamily is characterised by a conserved motor domain, which is the site of nucleotide turnover and the principal microtubule interaction domain. While the majority of kinesins move directionally along microtubules, members of the Kinesin-13 family do not support microtubule motility, these motors diffuse on microtubules and depolymerise microtubules from both ends [1–3]. There are four members of the mammalian Kinesin-13 family: KIF2A, KIF2B, KIF2C (also called MCAK) and KIF24. Their motor domain is conserved, but their N- and C-terminal flanking regions are divergent and provide specificity of function and targeting of activity [4]. The genome of most eukaryotes contains at least one Kinesin-13 [5]. Members of this family are suggested to be major regulators of microtubule length (reviewed in ref. [6]). Yeast, however, typically do not have a Kinesin-13, and instead, a member of the Kinesin-8 family likely takes up the role of microtubule length regulation [7].

Kinesin-13s play prominent roles in regulating the length of microtubules and are particularly important during meiosis and mitosis in higher eukaryotes. The first members of the Kinesin-13 family to be identified, KIF2A and MCAK, were found in mammals [8,9]. The Xenopus homologue of MCAK, XKCM1, was reported as a microtubule depolymerising kinesin with the ability to alter microtubule dynamics [3,10]. Depletion of XKCM1 from *Xenopus* extracts results in long microtubules and disorganised spindles, leading to chromosome misalignment [10]. Other Kinesin-13s are also shown to have microtubule destabilising activity. In Drosophila S2 cells and oocytes, depletion of the Kinesin-13, KLP10A, results in an excess of microtubules growing from the spindle, spindle disorganisation and chromosome misalignment [11–13]. KLP10A also controls centriole length [14]. During cell division in the *Caenorhabditis elegans* oocyte, the Kinesin-13, KLP-7 prevents ectopic cytoplasmic nucleation of microtubules, which can lead to multipolar spindle assembly and mitotic failure [15,16].

The most highly studied Kinesin-13 is the mammalian MCAK/KIF2C. Localisation of MCAK was first reported at the inner centromeres of chromosomes [8,10]. The centromeric pool of MCAK contributes to correct formation of kinetochore–microtubule attachments and to chromosome oscillations [17–19]. The activity of MCAK at centromeres is down-regulated by phosphorylation by Aurora B and other kinases [20–23]. Subsequently, MCAK was also found at the growing tips of microtubules [24], where it tracks growing microtubules in an EB-dependent manner [25,26]. The microtubule plus tip localisation of MCAK is also regulated by Aurora B kinase [27]. More recently, the Kinesin-8 KIF18B was found to mediate the plus tip targeting of MCAK [28]. The complex of MCAK and KIF18B regulates astral microtubule length and spindle bipolar assembly [28,29].

Another mammalian Kinesin-13, KIF2A, plays a critical role in neurons to regulate axonal pruning, essential for the correct establishment of the nervous system [30]. KIF2A localises primarily to centrosomes and spindle poles [4,31–33]. As with MCAK, KIF2A plays an important role in cell division and is suggested to be required for bipolar spindle formation [32,33]. KIF2B is absent or present at very low levels in most cell types, while moderately expressed in testes [34]. Its proposed role in regulation of microtubule dynamics and correction of microtubule–kinetochore mis-attachments is under debate, as the KIF2B depletion phenotype that results in monopolar spindles cannot be rescued [4,27]. Other studies, including the Mitocheck study, did not find any mitotic phenotype upon depletion of KIF2B [4,27,35]. KIF2B associates with Cep170 to target to the spindle [4]. The final mammalian Kinesin-13, KIF24, plays a role in regulation of the length of cilia and has a longer C-terminal region than the other mammalian Kinesin-13s [36,37].

In this review, we will describe the structural components of a typical Kinesin-13 and how these are adapted either to promote microtubule depolymerisation or to regulate the activity of a microtubule depolymerase.

## Domain structure of a Kinesin-13

**Figure 1.**
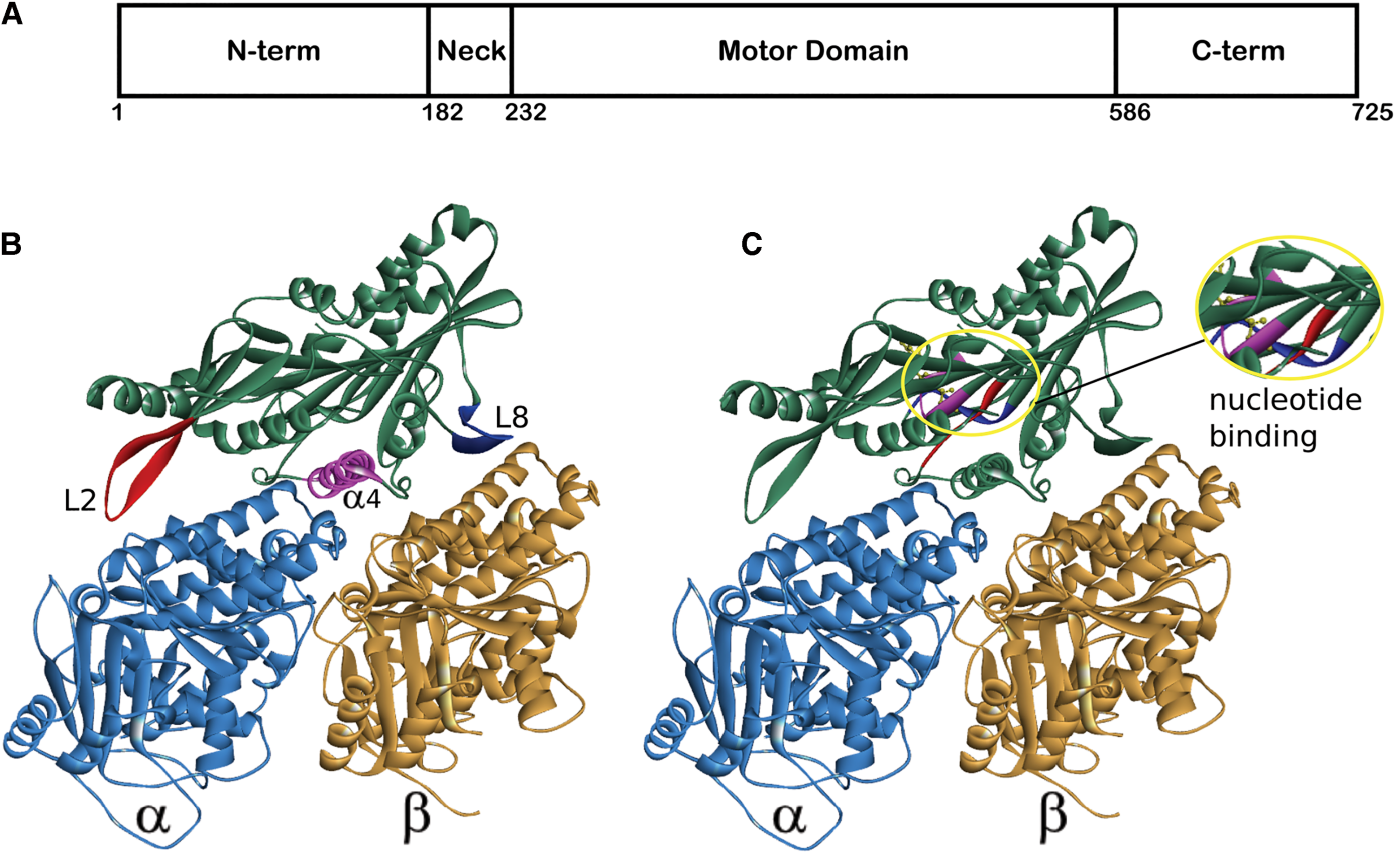
Typical domain layout and motor domain structure of a Kinesin-13. (**A**) The domain layout of a typical Kinesin-13, numbering is according to the sequence of the human homologue of MCAK/KIF2C. (**B**,**C**) Structure of the human Kinesin-13, KIF2C, in complex with an α/β-tubulin heterodimer (PDB: 5MIO) [42]. (**B**) The major pieces of secondary structure that defines the microtubule-binding interface are highlighted: Loop 2 (red), α4-helix (pink) and Loop 8 (blue). (**C**) The location of the nucleotide-binding site is shown inside the yellow oval, plus a magnified view of this region. The major nucleotide-binding motifs are highlighted: p-loop (pink), Switch I (blue) and Switch II (red).

In the typical Kinesin-13, the characteristic kinesin motor domain is positioned centrally in the primary sequence (Figure 1A). The most highly studied Kinesin-13, MCAK, has an N-terminal domain, followed by a positively charged region known as the neck, a centrally located motor domain and a C-terminal domain. This basic domain structure is also found in other members of the Kinesin-13 family.

## The motor domain

The motor domain is the domain that defines a member of the Kinesin superfamily [38]. It is both the nucleotide-binding site and the principle microtubule interaction domain. Motor domain-only truncation constructs of the Kinesin-13 MCAK are able to depolymerise microtubules [39], indicating that the Kinesin-13 motor domain alone has microtubule depolymerase activity.

### Microtubule-binding interface

**Figure 2.**
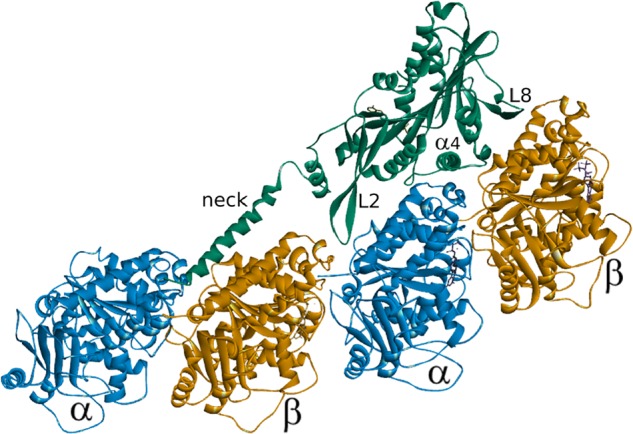
Structure of a complex of the human Kinesin-13, KIF2A (green), in a 1 : 2 complex with α/β-tubulin (blue and orange) (PDB: 6BBN) [44]. In addition to the major pieces of secondary structure that define the principle microtubule-binding interface, the location of the neck helix is shown interacting with the tubulin dimer on the minus-end side of the tubulin to which the motor domain is bound.

The major microtubule-binding face of the motor domain is composed of Loop 2, the α4-helix, Loop 12, the α5-helix and Loop 8 (Figure 1B). It has long been suggested that the microtubule-binding interface of the Kinesin-13 motor domain is adapted to interact more favourably with a curved conformation of tubulin rather than the straightened conformation present within the microtubule lattice [40]. A series of recently published structures of the complex between a Kinesin-13 motor domain and tubulin heterodimers confirm this suggestion [41–44]. In their structure of a 1:1 complex between the α/β-tubulin heterodimer and a motor domain-only construct of human KIF2C/MCAK fused to the ankyrin repeat protein DARPin, Wang et al. [42] highlight that the curvature at the intradimer tubulin interface is larger than that observed in similar complexes of tubulin with a Kinesin-1 motor domain: 14.7° compared with 11.6° in the same nucleotide state. Similarly, Ogawa et al. [43] compared their crystal structures of a mouse KIF2A motor domain purified from a complex with tubulin with structures of the unbound KIF2C motor domain. The major difference is the increased curvature of the tubulin-binding surface: superposition of the bound and unbound structures shows that the Loop 8 region is warped upward in KIF2C that had been bound to tubulin increasing the curvature of the binding interface. A recent structure of the human KIF2A motor domain in complex with tubulin shows a curvature of 12.1° at the α-/β-tubulin intradimer interface bound by the motor domain [44]. The authors also highlight curvature at both the interdimer interface (15.8°), mediated by the interaction of tubulin with Loop 2 and the neck region, and the intradimer interface of the tubulin prior to the motor domain-bound heterodimer (15.3°) mediated by interaction with the neck region (Figure 2). Benoit et al. [41] solved the structure of the Kinesin-13, KLP10A from *Drosophila melanogaster*, both in complex with curved and with straight tubulin by cryoEM. Here, they see an ∼12° curvature at both the intradimer and interdimer tubulin interface (24° per heterodimer) suggested to be induced by KLP10A binding.

All four structures confirm the orientation of the Kinesin-13 motor domain in relation to tubulin (Figure 1B). The α4-helix is seen to sit in the intradimer groove between α- and β-tubulin. The Loop 8 region is situated at the distal end of the β-tubulin subunit and the family specific long Loop 2 binds at the longitudinal interface between β- and α-tubulin: the interdimer groove [41–44]. The microtubule-binding face of the Kinesin-13 motor domain contains two family specific sequence motifs. These motifs are located in the α4 helix and in the β-hairpin that forms Loop 2 [40,45].

Each of the recent structures of the Kinesin-13 motor domain in complex with tubulin shows that the central axis of the tubulin-binding interface is the α4 helix, found in the intradimer groove (Figure 1B). The α4 helix contains the family specific highly conserved motif KECIR. Several of these residues are vital to the microtubule depolymerisation activity of MCAK [40,46] and recent work has shown that they are critical to the ability of MCAK to recognise and bind to the microtubule end [45]. Phosphorylation by Cdk1 at a location immediately C-terminal to the α4 helix has also been shown to impair microtubule end recognition [47]. The recent structures of the Kinesin-13 motor domain in complex with the tubulin heterodimer show that the residues K, E and R of this family specific motif make contact with the loop connecting H11 and H12 in α-tubulin. This places them in a position to sense conformational changes at the intradimer interface between α- and β-tubulin, which is the suggested mechanism of microtubule end recognition [41,42,44].

The β-hairpin that forms Loop 2 of the kinesin motor domain is significantly longer in members of the Kinesin-13 family than in other kinesins. In each of the recent structures of Kinesin-13–tubulin complexes, Loop 2 is found to interact at the α-tubulin side of the heterodimer in the interdimer groove and so would be oriented towards the minus-end of a microtubule (Figure 1B) [41–44]. This loop and the family specific KVD motif it contains are critical for microtubule depolymerisation activity [40,46]. The molecular mechanism underlying the importance of Loop 2 to the process of microtubule depolymerisation remains unclear. This region may simply contribute to the tubulin curvature that destabilises the microtubule structure, as is suggested by the structure of KIF2A in complex with tubulin (Figure 2), and/or play a more active role in directly severing longitudinal contacts between tubulin heterodimers.

### Second tubulin-binding site

The Kinesin-13, KLP10A, has been shown to have a secondary tubulin-binding site on the opposite side of the motor domain from the principal tubulin-binding interface [48]. This additional tubulin-binding site mediates the formation of oligomeric rings and spirals of tubulin observed by cryoEM of depolymerising microtubules. Residues that comprise this secondary microtubule-binding region differ in their electrostatic potential in the Kinesin-13 family relative to other kinesin families. In most kinesins, this region is negatively charged. However, in members of the Kinesin-13 family, it tends to be positively charged, which would facilitate an interaction with the negatively charged surface of microtubules. This second microtubule-binding region may have a physiological role in cell division, and disruption of this region in the *D. melanogaster* Kinesin-13, KLP10A, causes mitotic defects in cultured cells [48].

### Nucleotide-binding site

The nucleotide-binding site is formed from three sequence motifs typical of ATP-binding proteins (Figure 1C). These three highly conserved motifs are the p-loop (GQTGSGKT in Kinesin-13s), Switch I (NxxSSRSH) and Switch II (DLAGxER) [45]. The p-loop is a common phosphate-binding motif, which typically interacts with the β-phosphate of the nucleotide. The Switch I and Switch II motifs are responsible for sensing the phosphate state of the nucleotide and typically undergo significant conformational changes during the ATPase cycle of a kinesin to communicate the nucleotide status of the motor domain to the microtubule-binding interface. The Kinesin-13 MCAK has an ATP turnover cycle that is not typical among the Kinesin superfamily [49]. Rather than ADP dissociation acting as the rate-limiting step in the absence of tubulin, in the case of MCAK it is ATP cleavage that is rate-limiting under these conditions. The conformation of the nucleotide-binding site of MCAK in the absence of tubulin suggests why ATP cleavage is rate-limiting in the absence of tubulin. The nucleotide-binding pocket is in an open conformation in which the Switch loops are not close enough to form a salt bridge thought to be required for nucleotide cleavage [40]. Interaction with tubulin accelerates ATP cleavage by MCAK, suggesting that binding to tubulin causes a conformational change in the nucleotide-binding site that promotes ATP cleavage [49]. In agreement with this, the structure of a complex of KLP10A with curved tubulin shows that the nucleotide-binding pocket of the motor domain adopts the ‘closed’ conformation thought to represent the ATP hydrolysis competent state [41].

## Neck

A short region N-terminal to the motor domain is named the neck in Kinesin-13s (Figure 1A). The Kinesin-13 neck does not appear to play the same role the region of the same name found on the opposite side of the motor domain in translocating kinesins. Structures of isolated Kinesin-13 neck–motor domain constructs show that the neck region forms a helical structure [40]. Removal either of the entire neck region or of the positively charged residues contained in the neck region of MCAK/KIF2C indicates that the neck is crucial for maximal depolymerisation activity [39,50]. Protein engineering studies suggest that the neck enhances delivery of MCAK to the microtubule end by catalysing association with microtubules [39,51]. Recently published structures of the Kinesin-13 motor domain in a 1:2 complex with tubulin show the neck helix interacting with the β-subunit of the tubulin heterodimer on the microtubule minus-end side of the tubulin to which the motor domain is bound (Figure 2) [41,44]. The structure of the KIF2A neck–motor construct in complex with tubulin shows the neck helix stretched as far as the intradimer groove of the previous heterodimer and is suggested to be the cause of the 15.3° curvature observed for this heterodimer and the 15.8° bend observed at the interdimer interface (Figure 2). This localisation of the neck helix on the tubulin dimer prior to the one to which the motor domain is bound is suggested to explain the alternating pattern of motor domain decoration of microtubule protofilaments previously observed [52]. The presence of the neck region likely sterically inhibits the binding of a motor domain to two contiguous tubulin subunits in a protofilament.

## N-terminal and C-terminal domains

**Figure 3.**
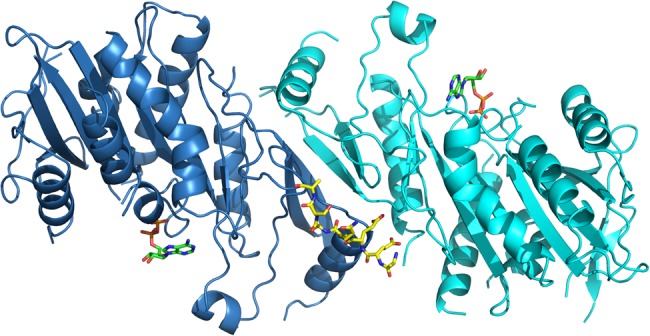
An MCAK motor domain dimer (cyan and blue) bound to a C-terminal peptide (yellow, stick representation) of MCAK (PDB: 4UBF). ADP is in green stick representation. Oxygen and nitrogen atoms are in red and blue, respectively. Phosphates are orange.

The non-motor regions are highly divergent within the Kinesin-13 family and allow for targeting and regulatory specificity. In the most highly studied Kinesin-13, MCAK, the non-motor regions not only facilitate the formation of a homodimer required for maximal microtubule depolymerisation activity [53] but also influence MCAK's activity via long-range interactions with the motor domain. The far C-terminal region of MCAK has been shown to inhibit microtubule-stimulated ATP turnover by the motor domain [54]. Recent work has shown that the C-terminal region interacts with the motor domain influencing the conformation of MCAK via these interactions [55–58]. In solution, the C-terminus of MCAK induces motor domain dimerisation. The crystal structure of the far C-terminus of MCAK bound to the motor domain shows that a peptide of the far C-terminal sequence binds at the interface between two MCAK motor domains away from both the microtubule-binding face and the ATP-binding pocket [55] (Figure 3). However, how the motors relate to each other in the context of full-length MCAK is currently not known. In a dimer of full-length MCAK molecules, the second C-terminal tail may be free to engage in other interactions. The far C-terminus of MCAK must be displaced from the motor domain to allow the motor to engage with the microtubule lattice, and thus, provide a means of regulation of depolymerisation activity. The C-terminus of KIF2A and KLP10A possess the same motif (EExxS) found at the far C-terminus of MCAK, indicating they too may be regulated through interactions between the motor and C-terminal domains [55]. FRET studies also found that the residues in the far C-terminus supporting the motor–tail interaction in the crystal structure were essential for the interaction with the motor domain and the C-terminus in the *Xenopus* homologue of MCAK in solution [56,57]. This interaction, controlled by mitotic phosphorylation, regulates MCAK activity with implications for spindle assembly and chromosome segregation [59].

The N-terminus of Kinesin-13s is highly divergent and defines targeting and functional specificity. The N-terminus of KIF2A targets mainly to centrosomes and weakly to the ends of kinetochore microtubules [4,60]. The N-terminal region is required for localisation of MCAK/KIF2C to the centromere and kinetochore [61,62]. Cellular localisation of MCAK/KIF2C has been shown to occur via association with other proteins. An interaction with Shugoshin 2 targets to kinetochores and, an interaction with EB proteins, via the N-terminal region of MCAK, targets to microtubule ends [24–26,63]. The N-terminus of KIF2B does not appear to specify localisation to a specific microtubule substructure but localises to microtubules. However, the C-terminus of KIF2B associates with another microtubule-binding protein Cep170, which targets KIF2B to the spindle [4]. Swapping the N-termini of KIF2A, KIF2B and MCAK/KIF2C changes the targeting of the motor domain and the associated depolymerase activity [4].

## Summary and questions

The Kinesin-13 motor domain alone possesses potent microtubule depolymerising activity, indicating that the motor domain of this Kinesin family is specifically adapted for microtubule depolymerisation. The molecular characteristics that confer specialist depolymerising activity to the Kinesin-13 family are not fully understood. However, the structures and mutational studies described here indicate that the tubulin-binding interface is specifically adapted to recognise the microtubule end. This adaptation likely confers an ability to sense curved conformations of tubulin that exist at or close to microtubule ends due to increased conformational freedom not available to subunits embedded in the lattice. Family specific sequence motifs found on the tubulin-binding interface confer crucial variation in the interaction of the Kinesin-13 motor domain with tubulin compared with the rest of the superfamily. The α4 helix is essential for the ability to distinguish the microtubule end from the microtubule lattice, likely by sensing curvature at the intradimer interface. The precise role of the family specific Loop 2 remains unclear. It is possible that it performs a similar role to the α4 helix, recognising the microtubule end by sensing curvature at the interdimer rather than the intradimer interface. Microtubule end recognition is crucial to the activity of Kinesin-13s and kinesins that regulate microtubule dynamics in general. It will be interesting to discover if other microtubule-regulating kinesins use the same means of end recognition and how the balance between binding to tubulin embedded in the lattice and microtubule end recognition is obtained by kinesins that possess both translocating and regulating activity, for example the Kinesin-8 family.

The neck region is required for maximal microtubule depolymerisation activity. Mutational studies have shown that for the Kinesin-13 MCAK, this region is critical to delivery to the microtubule end via diffusion on the lattice [51]. Recent structures of Kinesin-13 neck–motor constructs suggest a possible additional role at the site of depolymerisation as the neck helix is seen to bind across tubulin subunits [41,44]. These structures suggest that the full part played by the Kinesin-13 neck in microtubule depolymerisation is yet to be fully uncovered.

The non-motor regions of the Kinesin-13 family regulate their cellular localisation and therefore provide cellular functional specificity to the various family members. However, these regions clearly have a further role via the formation of long-range intramolecular interactions that alter the conformation of full-length Kinesin-13s. These interactions between the N- and C-terminal domains, and the motor and neck domains regulate the interaction of Kinesin-13s with microtubules, similar to other kinesins such as CENP-E, Kinesin-1 and KIF17 (reviewed in ref. [64]). Another role of the Kinesin-13 non-motor regions is to mediate the formation of the homodimers in which Kinesin-13s function in cells. Since monomeric constructs of Kinesin-13s can display similar rates of microtubule depolymerisation to full-length versions *in vitro*, it remains something of a mystery why these kinesins act as dimers in the physiological setting. One hypothesis is that the presence of two motor domains in the active molecule gives a degree of processivity to the removal of tubulin subunits from the microtubule end. However, the reason behind the requirement for dimerisation remains to be fully understood.
